# Sulforaphane metabolites inhibit migration and invasion via microtubule-mediated Claudins dysfunction or inhibition of autolysosome formation in human non-small cell lung cancer cells

**DOI:** 10.1038/s41419-019-1489-1

**Published:** 2019-03-15

**Authors:** Zhongnan Zheng, Kai Lin, Yabin Hu, Yan Zhou, Xiaoyan Ding, Yalin Wang, Wei Wu

**Affiliations:** 1 Department of Biochemistry and Molecular Biology, School of Basic Medical Sciences, Capital Medical University, Beijing, China; 2 Beijing Key Laboratory for Tumor Invasion and Metastasis, Institute of Brain Tumor, Beijing Institute for Brain Disorders, Beijing, China; 3 Cancer Center of Beijing Ditan Hospital Affiliated to Capital Medical University, No. 10, Xitoutiao, You An Men Wai Avenue, Feng Tai District, Beijing, 100069 China

## Abstract

Both sulforaphane-cysteine (SFN-Cys) and sulforaphane-*N*-acetyl-l-cysteine (SFN-NAC) inhibited cancer migration and invasion, but the underlying mechanisms were not clear. Here we uncovered via tissue microarray assay that high expression of invasion-associated Claudin-5 was correlated to malignant grades in human non-small cell lung cancer (NSCLC) cells. Further, SFN-Cys (10 µM) induced the accumulated phosphorylation of ERK1/2, leading to downregulation of Claudin-5 and upregulation of Claudin-7, and the decrease of Claudin-1 in SK-1 cells and increase of Claudin-1 in A549 cells; knockdown of Claudin-5 significantly reduced invasion, whereas knockdown of Claudin-7 increased invasion; knockdown of Claudin-1 reduced invasion in SK-1 cells, whereas it increased invasion in A549 cells, indicating that SFN-Cys regulated Claudins and inhibited invasion depending on Claudin isotypes and cell types. Furthermore, immunofluorescence staining showed that SFN-Cys triggered microtubule disruption and knockdown of α-tubulin downregulated Claudin-1, 5, and 7, and inhibited migration and invasion, indicating that microtubule disruption contributed to invasive inhibition. Co-immunoprecipitation and confocal microscopy observation showed that SFN-Cys lowered the interaction between α-tubulin and Claudin-1 or 5, or 7. Meanwhile, Western blotting and immunofluorescence staining showed that SFN-NAC (15 µM) downregulated α-tubulin resulting in microtubule disruption; knockdown of α-tubulin increased SFN-NAC-induced LC3 II accumulation in SK-1 cells. Combined with the inhibitor of autolysosome formation, Bafilomycin A1 (100 nM), SFN-NAC inhibited invasion via accumulating LC3 II and blocking formation of autolysosome. Further, SFN-NAC upregulated microtubule-stabilizing protein Tau; knockdown of Tau reduced LC3 II/LC3 I inhibiting migration and invasion. These results indicated that SFN-Cys inhibited invasion via microtubule-mediated Claudins dysfunction, but SFN-NAC inhibited invasion via microtubule-mediated inhibition of autolysosome formation in human NSCLC cells.

## Introduction

Vegetable-derived sulforaphane (SFN) inhibits carcinogenesis and induces apoptosis in a variety of cancer cells^1–4^. Both SFN-cysteine (SFN-Cys) and SFN-*N*-acetyl-l-cysteine (SFN-NAC), as the metabolites of SFN, have longer retention time in circulation and were rich in the lung^5^. We previously reported that SFN-Cys inhibited migration and invasion via regulating invasion-associated proteins in couple of cancer cells^6–8^. Invasion-associated proteins, Claudins (1, 5, and 7), were demonstrated to correlate to cancer migration and invasion^9–11^. Also, we demonstrated that SFN-NAC (30 µM) induced apoptosis via microtubule disruption-mediated inhibition of autolysosome formation in non-small cell lung cancer (NSCLC) cells^12^. As cell proliferation and death affect cell motility, either SFN-Cys or SFN-NAC might inhibit migration and invasion via regulating either Claudins or microtubule-mediated autophagy.

Microtubule proteins α-tubulin and β-tubulin, microtubule-stabilizing proteins Tau, MAP1, MAP2, MAP4, and LC3, and microtubule-destabilizing protein Stathmin-1 contributed to cell motility. Microtubule moves by increasing its extension at the one end and shortening at the other end. Anti-cancer drugs paclitaxel and vinblastine inhibited tumor invasion and metastasis by producing disequilibrium of microtubule dynamics^13^. Studies showed that SFN analogs covalently bind to α-tubulin to cause microtubule depolymerization^14^. Simultaneously, we uncovered that SFN-Cys (20 µM) downregulated the expression of α-tubulin via phosphorylated ERK1/2 resulting in disrupted microtubules in NSCLC cells^15^. A couple of studies showed that the accumulation of phosphorylated ERK1/2 contributed to cell apoptosis and the inhibition of invasion^6,7^. Microtubule changed cell motility via regulating a variety of proteins, such as Claudins, E-cadherin, integrin, CD44v6, etc. Human Claudin family has at least 27 members, which are 22–27 kDa adhesion molecules^16^. Claudin-1 overexpression is associated with advanced clinical stage and invasive characteristics of oral squamous cell carcinomas^17^. Claudin-1, 2, 3, and 5 have the potential to interact with the MT1-MMP (matrix metalloproteinase) and this interaction might promote cell motility via degradation of the extracellular matrix^18–20^. Claudin-1 was upregulated by autophagy leading to p62 degradation under starvation^21^. Further, Claudin-1 might increase drug resistance in NSCLC cells by inducing autophagy^22^. Conversely, Claudin-1 might inhibit invasion in A549 cells^23^. Claudin-5 increased cell motility in breast cancer and increased expression of Claudin-7 reduced cell invasion in couple of cancers^24,25^. Here we aim at characterizing why Claudins exhibit distinct functions in cell motility in terms of different cell types. Claudins span the membrane four times, with cytosolic N- and C-terminal domains and two extracellular loops. This structure gives Claudins the potential to mediate interactions between the intracellular and extracellular molecules. The cytosolic C-terminal domain of Claudins contains a PDZ-binding domain, which is known to bind the cytoplasmic proteins ZO-1, ZO-2, and ZO-3, thus linking the tight junction to the cytoskeleton^26^. Recent report showed that Claudin-11 interacted with α-tubulin promoting cell migration^27^, indicating that microtubule might act as a scaffold to regulate Claudins function, autophagy, and invasion.

In addition to α-tubulin and β-tubulin, Tau also involves microtubule polymerization; once α-tubulin and β-tubulin heterodimers form microtubule, Tau perpendicularly binds to fibril filaments, thereby reducing the flexibility and increasing the stability of microtubules, maintaining the balance of microtubule dynamics^28^, participating in the regulation of the transport of materials^29^. Studies showed that Tau was highly expressed in several chemotherapy-resistant patients^30^; thus, the expression of Tau was commonly regarded as an indicator for drug resistance^31^. Overexpression of Tau promoted autophagy and inhibited cell apoptosis through multiple mechanisms including the p53-mediated endogenous apoptotic pathway^32^. However, the roles of Tau in cancer migration and invasion have not been elucidated; thus, characterization of Tau molecular signaling and function might be helpful to find the correlations of microtubule dynamics to autophagy and invasion.

Autophagy is an intracellular lysosomal degradation process to maintain homeostasis by mediating degradation of damaged organelles, and misfolded proteins; microtubule dynamics regulated autophagy^33^. First, as one of autophagic fluxes, the microtubule-associated protein light chain 3 II (LC3 II) was upregulated, processed, and recruited to autophagosomes, then LC3 II moved along microtubule to link to lysosome^34^. Autolysosomes are generated through fusing the outer membranes of the autophagosomes to lysosomes and further LC3 II is degraded; the inhibitor of autolysosome formation Bafilomycin A1 is able to stop the fusion^35^. Microtubule acted as a scaffold and might have a trafficking role in the formation of autophagosomes and autolysosomes. We reported that higher doses of SFN metabolites induced microtubule disruption, inhibiting the fusion of autophagosome to lysosome^12^. Higher concentration of SFN metabolites, which caused apoptosis^36^, could not be used for invasion study, because we do not know whether the number of decreased invasive cells resulted from invasion inhibition; it might result from cell death. Therefore, we will use lower concentrations of SFN metabolites, which do not induce apoptosis, to investigate the mechanisms inhibiting invasion.

Taken together, we will make sure how SFN-Cys inhibits invasion via microtubule-mediated deregulation of Claudin-1, 5, and 7, and how SFN-NAC inhibits invasion via α-tubulin and Tau-mediated autophagy, respectively. These studies will help us find out novel anti-cancer targets to develop high-efficiency therapeutics.

## Materials and methods

### Reagents

SFN-Cys and SFN-NAC were purchased from Santa Cruz Biotechnology (CA, USA). Lipofectamine^TM^ RNAiMAX was purchased from Invitrogen-Life Technologies (CA, USA). Anti-ERK1/2 (1:1000), anti-phospho-ERK1/2 (1:1000), and phosphorylated ERK1/2 inhibitor PD98059 were purchased from Cell Signaling Technology, Inc. (Shanghai, China). Anti-Claudin-1 (1:2000) was purchased from Abcam (MA, USA), anti-Claudin-5 (1:1000) was purchased from Santa Cruz (CA, USA), and anti-Claudin-7 (1:100) was purchased from Sangon Biotech, Ltd (Shanghai, China). Anti-LC3 was purchased from Cell Signaling Technology (CO, USA). Anti-Tau (Tau46) and anti-α-tubulin (B-7) were purchased from Santa Cruz (TX, USA). Autophagy inhibitor 3-Methyladenine (3-MA) was bought from Sigma (Hongkong, China) and Bafilomycin A1 (Baf-1) was from Selleck (Shanghai, China).

### Cell culture, viability assay, and transfection

Human NSCLC A549 and SK-1 cells were purchased from the Cell Resource Center, Peking Union Medical College. Cells were incubated in Dulbecco’s modified Eagle’s medium (DMEM)/F-12 culture medium with 10% fetal bovine serum (FBS), 100 U/ml penicillin and streptomycin at 37 °C in a humidified incubator containing 5% CO_2_. Cell viability was evaluated via cell proliferation assay kit as described in the specifications (WI, USA). For gene knockdown, negative control small interfering RNA (siRNA) (5′-UUCUCCGAACGUGUCACGUTT-3′) and Tau siRNA (5′-CCGCCAGGAGUUCGAAGUGAU-3′), Claudin-1 siRNA (5′-GCAUGGUAUGGCAAUAGAA-3′), Claudin-5 siRNA (5′-CCAACAUUGUCGUCCGCGATT-3′), Claudin-7 siRNA (5′-AUUAGGGCUCGAGUGGCCUGCAAGG-3′), and α-tubulin siRNA (5′-AGAUGUCAAUGCUGCCAUU-3′) sequences were designed in reference to the literatures^37–41^. Cells were plated in six-well plates at a density of 1 × 10^6^ per well and cultured for 24 h; the medium was replaced with fresh medium containing 10% FBS when cells reach ~70% confluency. Then cells were transfected with the corresponding siRNA or negative control siRNA (30 pmol/well) by Lipofectamine^TM^ RNAiMAX and were cultured for 24 h continuously. Cells were cultured for 24 h, respectively. After 48 h transfection, cells were treated with SFN-Cys or SFN-NAC for 24 h and collected for further assays.

### Scratch assay

Wound scratch healing assay was performed to detect the ability of cell migration. Cells were seeded at a density of 2 × 10^6^ cells per well in a six-well plate and incubated overnight. Three parallel thin “wounds” and one vertical “wound” were scratched by a yellow pipette tip when cells reach ~100% confluency. The cells were washed with phosphate-buffered saline (PBS) and then incubated in serum-free medium with serial concentrations of SFN-Cys or SFN-NAC for 24 h. The images were captured by a phase-contrast microscope (Leica) at 0 and 24 h, and the wound areas were calculated by the Image-pro plus 6.0.

### Invasion assay

Matrigel basement membrane matrix was diluted with serum-free DMEM/F-12 medium to 2 mg/mL and then plated onto Transwell chamber, which was placed into a 24-well plate. These Transwell chambers were rehydrated at 37 °C for 1 h before seeding cells. A total of 2 × 10^4^ cells were added to the upper chamber with serum-free DMEM/F-12 medium and 500 µL DMEM/F-12 medium containing 10% FBS was added to the lower chamber. After incubation with different concentrations of SFN-Cys or SFN-NAC for 24 h, the cells were fixed with 100% methanol for 20 min and then stained with 0.5% crystal violet solution for 30 min. The cells were washed three times with PBS and the cells on the top of upper chamber were removed gently with a cotton swab. The invasive cells were observed in at least five to six randomly selected fields per well under microscope. The data were analyzed by ImageJ.

### Western blotting

Cells were treated with SFN-Cys or SFN-NAC and then lysed with RIPA lysis buffer. Cell lysate was centrifuged and the supernatant was collected. Total protein concentrations were determined by BCA Protein Assay Kit. The procedures were carried out according to the previous experimental method^36^.

### Tissue microarray immunohistochemical assay

Human lung adenocarcinoma and lung squamous carcinoma tissue arrays containing 150 dotted tumor and adjacent tissues from 75 patients with different pathologic grades were purchased from Shanghai Biochip (Shanghai, China). The immunohistochemistry stain was done with human-specific anti-Claudin-1, 5, and 7 combined with the UltraSensitive^TM^ S-P detection kit (Fuzhou, China). The protocol was derived from a published paper^12^.

### Bioinformatics analysis

We searched the GEPIA Database to find the possible correlation between survival rate and invasion-related proteins including Claudin-1, Claudin-5, and Claudin-7^42^. We also searched the version 10.5 of STRING database to find the interaction among Claudins and microtubule proteins^43^.

### Immunofluorescence staining and confocal microscopy observation

Cells were seeded in 35 mm cover glass-bottom dishes at a density of 1 × 10^5^ cells/dish and incubated for 24 h, then treated with 10 µM SFN-Cys or 15 µM SFN-NAC (these concentrations were determined by cell proliferation assay in the preliminary studies) for 24 h. These cells were fixed with 1% paraformaldehyde for 15 min. The cells were washed three times with PBST (PBS with Tween-20) and permeabilized with − 20 °C methanol for 10 min at room temperature. After blocking with PBS containing 1% bovine serum albumin and 0.1% Triton X-100 for 1 h, the cells were incubated overnight at 4 °C with the corresponding primary antibodies. The cells were washed three times with PBST and incubated with the fluorescence-labeled secondary antibody for 1 h at room temperature. After washing with PBST three times, the cells were stained with DAPI (4′,6-diamidino-2-phenylindole). Fluorescence images were collected under a laser scanning confocal microscope (Olympus FV1000; Olympus Corp., Tokyo, Japan).

### Co-immunoprecipitation assay

The treated cells were washed with ice-cold PBS and then lysed on ice via nondenaturing lysis buffer supplemented with protease inhibitors cocktail. The monoclonal anti-α-tubulin or anti-Claudin-1 or 5, or 7, were added to the protein lysates, respectively, incubated overnight at 4 °C. The complexes were pulled down with protein A/G agarose for 3 h and the proteins were isolated by centrifuging and boiling for 5 min. Western blotting was used to recognize the conjugated proteins.

### Transmission electron microscopy observation

SK-1 cells were treated with 15 µM SFN-NAC for 24 h, then collected and washed with PBS for two times, and fixed with 3% glutaraldehyde at 4 °C for 2 h. After washing with PBS three times, the sample was fixed in 1% osmium tetroxide for 1 h. Samples were dehydrated through a series of concentrations of ethanol, and infiltrated and embedded in a 1:1 mixture of acetone and Epon-812 resin for 30 min. The samples were infiltrated in Epon-812 for 2 h, cut into ultrathin sections with a knife, and positioned on 200-mesh copper grids. Sections were stained with Uranium acetate for 30 min and then stained with Lead nitrate for 20 min. The sections were then observed and photographed with a transmission electron microscope (JEM-1400Plus, JEOL, Ltd, Tokyo, Japan).

### Autophagy assay

Transmission electron microscopy was used to view autophagosomes, autolysosome, etc. LC3 II/LC3 I was determined to be an indicator of autophagy via Western blotting. Immunofluorescence staining and confocal microscopy was used to observe the number of LC3 puncta. Bafilomycin A1 (100 nM) was used to inhibit the fusion of autophagosome to lysosome and 3-MA (2.5 mM) was applied to repress the initiation of autophagy.

### Statistical analysis

Data were expressed as mean ± SD from three independent experiments. Paired data were evaluated by Student’s *t*-test. Two-way analysis of variance was used to determine statistical significance. *P* ≤ 0.05 was considered statistically significant. All statistical analyses were performed by SPSS version 19.0.

## Results

### SFN-Cys inhibited cell proliferation and invasion

**Fig. 1:**
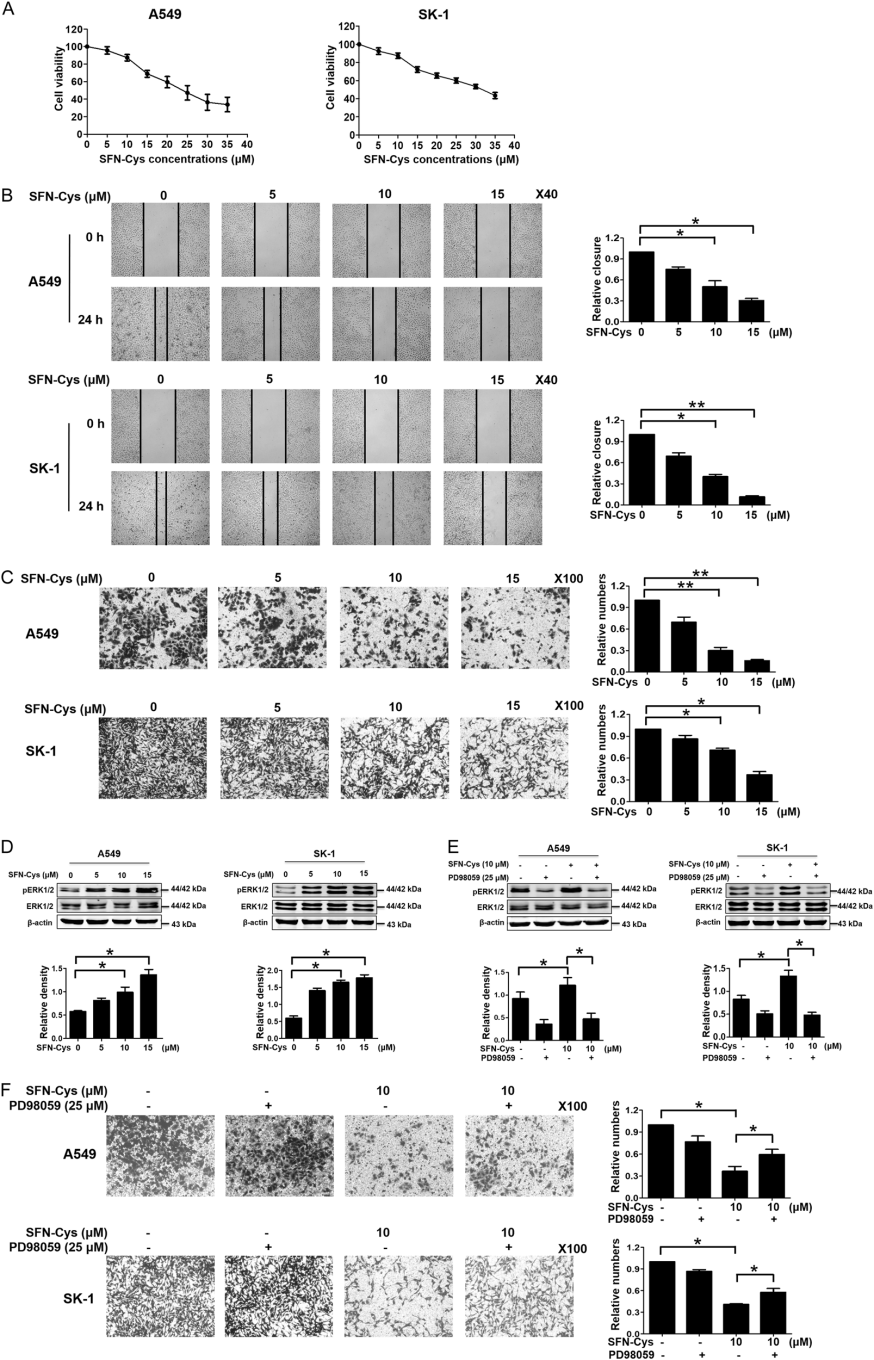
SFN-Cys inhibited migration and invasion via sustained ERK1/2 phosphorylation. **a** Cells were treated with increasing concentrations of SFN-Cys for 24 h, then cell viability was determined by Cell Proliferation Assay Kit and presented as the percentages vs. the control. **b** Cells were scratched and treated with SFN-Cys at the indicated concentrations for 24 h. An image of the wound closure area was captured at × 40 magnification and measured with ImageJ software. **c** The cells (1 × 10^5^) were seeded in 24-well invasion chambers and treated with increasing doses of SFN-Cys. Cells were stained with crystal violet, images were taken at ×100 magnification and analyzed. **d** Western blotting was used to analyze pERK1/2 expression. **e** Cells were pretreated with PD98059 (25 µM) for 30 min, then treated with SFN-Cys (10 µM) for 24 h. The expression of phosphorylated ERK1/2 was analyzed by Western blotting. **f** Cells were treated with phosphorylated ERK1/2 inhibitor PD98059 (25 µM) for 30 min, then 10 µM of SFN-Cys was added to the medium for 24 h. The invaded cells were counted. *P < 0.05, **P < 0.01. Data were shown as means ± SD (*n* = 3)

Cells were treated with increasing concentrations of SFN-Cys (0, 5, 10, 15, 20, 25, 30, and 35 µM) for 24 h. Results showed that cell viability was reduced significantly in a dose-dependent manner, indicating that SFN-Cys inhibited cell proliferation (Fig. 1a). Here, SFN-Cys (10 µM) inhibited cell growth but did not reduce cell number significantly and 10 µM was the optimal concentration for migration and invasion assay. The wound areas of the cells were observed via scratch assay under a microscope at 0 h and 24 h. Results showed that SFN-Cys significantly decreased cell migration in both A549 and SK-1 cells (Fig. 1b). After cells were treated with different concentrations of SFN-Cys (0, 5, 10, and 15 µM), these results showed that cell invasion was significantly decreased in a dose-dependent manner (Fig. 1c). Once the cells were treated with serial concentrations of SFN-Cys (0, 5, 10, and 15 µM) for 24 h, Western blotting showed that the expression of phosphorylated ERK1/2 was significantly enhanced (Fig. 1d). Phosphorylated ERK1/2 inhibitor, PD98059 (25 µM), reduced the expression of phosphorylated ERK1/2 and reversed the inhibition of invasion by SFN-Cys (Fig. 1e, f). Therefore, SFN-Cys inhibited cell invasion via sustained phosphorylation of ERK1/2.

### Claudins were expressed differentially in NSCLC tissues and SFN-Cys regulated Claudins via phosphorylated ERK1/2

**Fig. 2:**
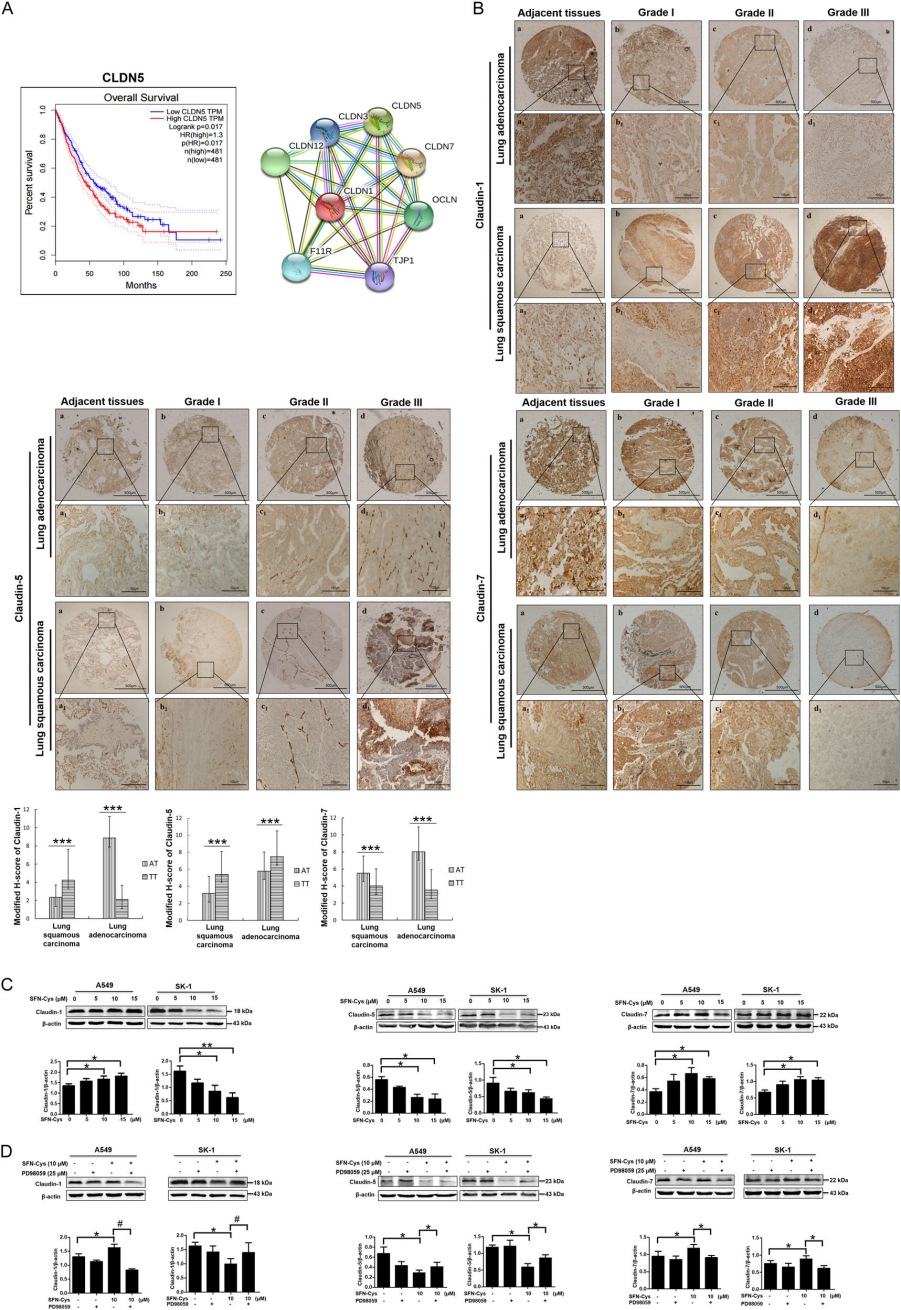
SFN-Cys regulated Claudin-1, 5, and 7 in A549 and SK-1 cells via phosphorylated ERK1/2. **a** Survival analysis in Claudin-5-expressed patients via GEPIA database and the interactions among Claudins and related partners were analyzed via the String net. (CLDN1: Claudin-1; CLDN5: Claudin-5; CLDN7: Claudin-7; TJP1: ZO-1; OCLN: Occludin; F11R: Junction adhesion molecule A) were shown. **b** Expressions of Claudin-1, 5, and 7 were evaluated in the microarray tissues with pathological grades of lung adenocarcinoma and lung squamous carcinoma. A549 cells belong to lung adenocarcinoma and SK-1 cells belong to lung squamous carcinoma. IHC of Claudin-1, 5, and 7 in adjacent and cancer tissues with three malignant grades were used (AT: adjacent tissues; TT: tumors tissues). Magnification × 200, Scale bars: 100 µm. **c** Western blotting was used to analyze Claudin-1, 5, and 7 expression in the cells treated with SFN-Cys at the indicated concentrations. **d** Cells were pretreated with PD98059 (25 µM) for 30 min, then treated with SFN-Cys (10 µM) for 24 h. The expressions of Claudin-1, 5, and 7 were analyzed by Western blotting. *P < 0.05; **P < 0.01; ***P < 0.001. Data were shown as means ± SD (*n* = 3)

Survival analysis showed that the patients with low expression of Claudin-5 had higher survival percentage, indicating that Claudin-5 might be a tumor promoter. STRING database showed that Claudin proteins interacted with tight junction proteins (Fig. 2a). Tissue microarray assay in human lung adenocarcinoma and lung squamous carcinoma showed that Claudin-5 was weakly stained in adjacent tissues, whereas darkly stained in tumor tissues, but Claudin-7 staining showed the opposite results. Interestingly, Claudin-1 was highly stained in lung squamous carcinoma and slightly stained in lung adenocarcinoma compared with adjacent tissues (Fig. 2b). The correlation of Claudin-1 expression to clinical staging and Claudin-5 expression to pathological grading of lung squamous carcinoma samples were also calculated by H-scores (Tables 1, 2 and 3). Western blotting showed that the expression level of Claudin-1 was significantly elevated in A549, but reduced in SK-1 cells with the increasing concentrations of SFN-Cys (Fig. 2c). Western blotting showed that PD98059 reversed the expression of Claudin-1 triggered by SFN-Cys (Fig. 2d). These results implied that Claudin-1 is the downstream effector of phosphorylated ERK1/2 in both A549 and SK-1 cells. Similarly, Western blotting showed that SFN-Cys downregulated Claudin-5 (Fig. 2c) and PD98059 reversed the effect (Fig. 2d), indicating that SFN-Cys decreased the expression of Claudin-5 via phosphorylation of ERK1/2. Western blotting also showed that Claudin-7 was upregulated by SFN-Cys in a dose-dependent manner (Fig. 2c) and PD98059 weakened the upregulation of Claudin-7 (Fig. 2d), indicating that activated ERK1/2 contributed to Claudin-7 upregulation.

**Table 1:** Correlation of Claudin-1 expression to clinicopathological characteristics of lung cancer patients

Variable	Lung squamous carcinoma				Variable	Lung adenocarcinoma			
	All patients	Low	High	*P*-value		All patients	Low	High	*P*-value
Gender				0.401	Gender				0.113
Male	71	38	33		Male	39	31	8	
Female	4	3	1		Female	35	22	13	
Age (years)				0.61	Age (years)				0.255
≤60	35	23	12		≤60	34	26	8	
>60	40	24	16		>60	41	28	13	
Differentiation				0.204	Differentiation				0.368
I	6	4	2		I	14	10	4	
II	44	24	20		II	42	28	14	
III	25	19	6		III	19	16	3	
Staging				0.004*	Staging				0.781
IA	9	7	2		IA	14	9	5	
IB	14	3	11		IB	23	20	3	
IIA	13	8	5		IIA	14	9	5	
IIIB	10	6	4		IIB	4	3	1	
IIIA	22	19	3		IIIA	11	8	3	
IIIB	2	0	3		IIIB	3	1	2	
IV	5	3	2		III	2	1	1	
					IV	4	3	1	

Low (score 0–4), High (score 5–12)

*P < 0.05 was defined as statistically significant

**Table 2:** Correlation of Claudin-5 expression to clinicopathological characteristics of lung cancer patients

Variable	Lung squamous carcinoma				Variable	Lung adenocarcinoma			
	All patients	Low	High	*P*-value		All patients	Low	High	*P*-value
Gender				0.084	Gender				0.972
Male	71	31	40		Male	39	11	28	
Female	4	0	4		Female	35	10	25	
Age (years)				0.975	Age (years)				0.432
≤60	35	15	20		≤60	34	8	26	
>60	40	17	23		>60	41	13	28	
Differentiation				0.036^*^	Differentiation				0.155
I	6	4	2		I	14	1	13	
II	44	20	24		II	42	14	28	
III	25	8	17		III	19	6	13	
Staging				0.597	Staging				0.216
IA	9	3	6		IA	14	2	12	
IB	14	5	9		IB	23	4	19	
IIA	13	6	7		IIA	14	5	9	
IIB	10	6	4		IIB	4	2	2	
IIIA	22	11	16		IIIA	11	4	7	
IIIB	2	1	1		IIIB	3	1	2	
IV	5	4	1		III	2	0	2	
					IV	4	3	1	

Low (core 0–4), High (score 5–12)

*P < 0.05 was defined as statistically significant

**Table 3:** Correlation of Claudin-7 expression to clinicopathological characteristics of lung cancer patients

Variable	Lung squamous carcinoma				Variable	Lung adenocarcinoma			
	All patients	Low	High	*P*-value		All patients	Low	High	*P*-value
Gender				0.676	Gender				0.63
Male	71	46	25		Male	39	27	12	
Female	4	3	1		Female	35	26	9	
Age (years)				0.364	Age (years)				0.193
≤60	35	21	14		≤60	34	27	7	
>60	40	28	12		>60	41	27	14	
Differentiation				0.67	Differentiation				0.51
I	6	3	3		I	14	11	3	
II	44	30	14		II	42	28	14	
III	25	16	9		III	19	15	4	
Staging				0.316	Staging				0.214
IA	9	8	1		IA	14	7	7	
IB	14	9	5		IB	23	15	8	
IIA	13	7	6		IIA	14	12	2	
IIB	10	7	3		IIB	4	3	1	
IIIA	22	12	10		IIIA	11	8	3	
IIIB	2	1	1		IIIB	3	3	0	
IV	5	5	0		III	2	2	0	
					IV	4	4	0	

Low (score 0–4), High (score 5–12)

*P < 0.05 was defined as statistically significant

### Claudins expression regulated invasion depending on cancer cell types

**Fig. 3:**
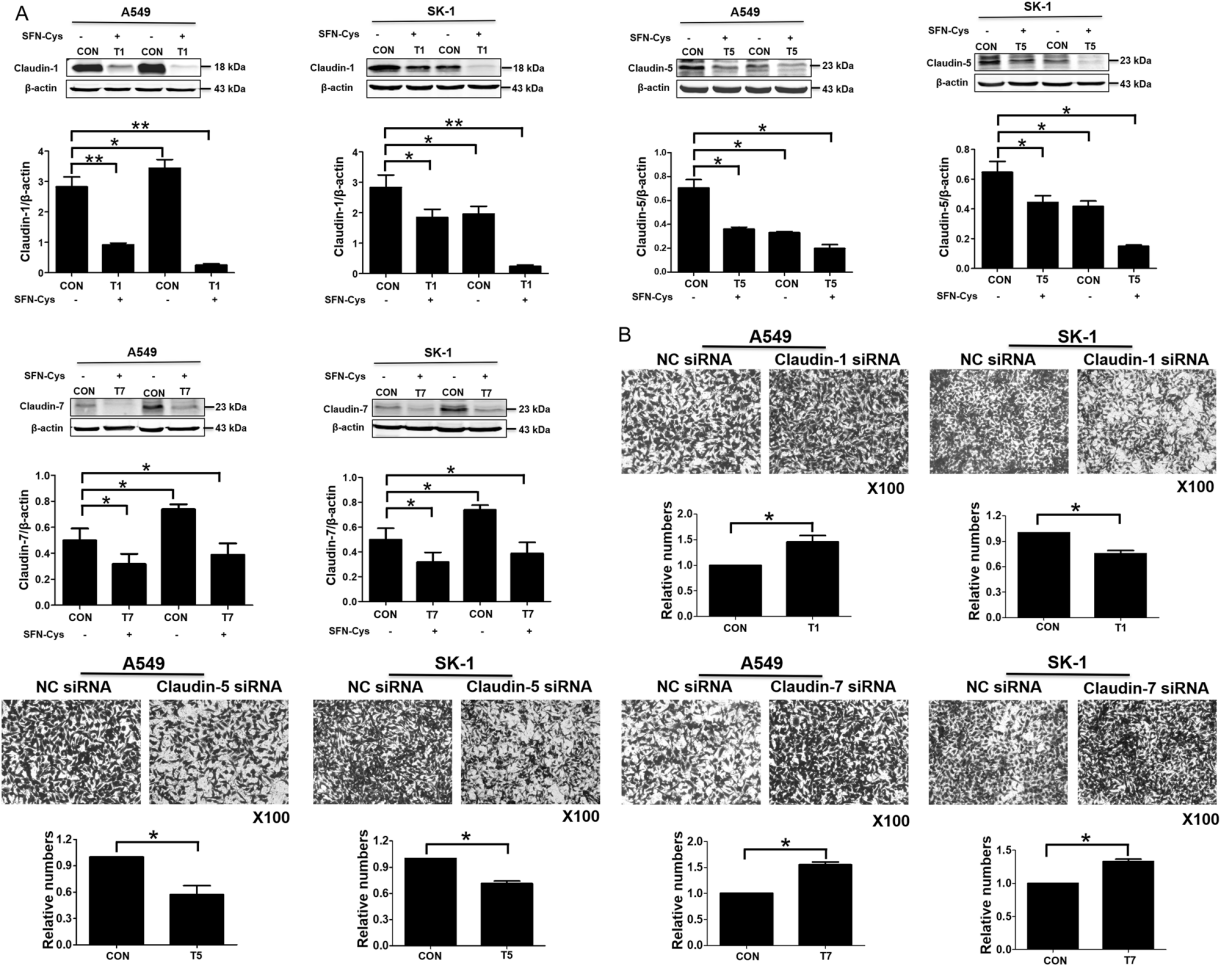
Knockdown of Claudin-1, 5, and 7 suppressed significantly migration and invasion in NCSLC cells. **a** Cells were transfected with normal control (NC) siRNA or Claudin-1, 5, and 7 siRNAs (Con: control; T1: transfection Claudin-1; T5: transfection Claudin-5; T7: transfection Claudin-7). After transfection for 48 h, the cells were seeded in six-well plates and treated with SFN-Cys for 24 h. Then the cells lysates were collected and the expressions of Claudin-1, 5, and 7 were analyzed by Western blotting. **b** Both A549 and SK-1 cells transfected with NC siRNA or Claudin-1, 5, and 7 siRNA were seeded in 24-well invasion chambers to detect invasion. *P < 0.05; **P < 0.01. Data were shown as means ± SD (*n* = 3)

Claudin-1, 5, and 7 siRNAs were used successfully to knock down the expression of those genes, respectively (Fig. 3a). Results showed that knockdown of Claudin-1 resulted in a significant increase of invasion in A549 cells and a significant decrease of invasion in SK-1 cells. Similarly, knockdown of Claudin-5 significantly decreased invasion and knockdown of Claudin-7 significantly increased invasion (Fig. 3b). These data indicated that Claudins expression might affect invasion depending on Claudin isotypes and cell types.

### Knockdown of α-tubulin regulated the expression of Claudin-1, 5, and 7, and SFN-Cys decreased the binding of Claudin-1, 5, and 7 to α-tubulin

**Fig. 4:**
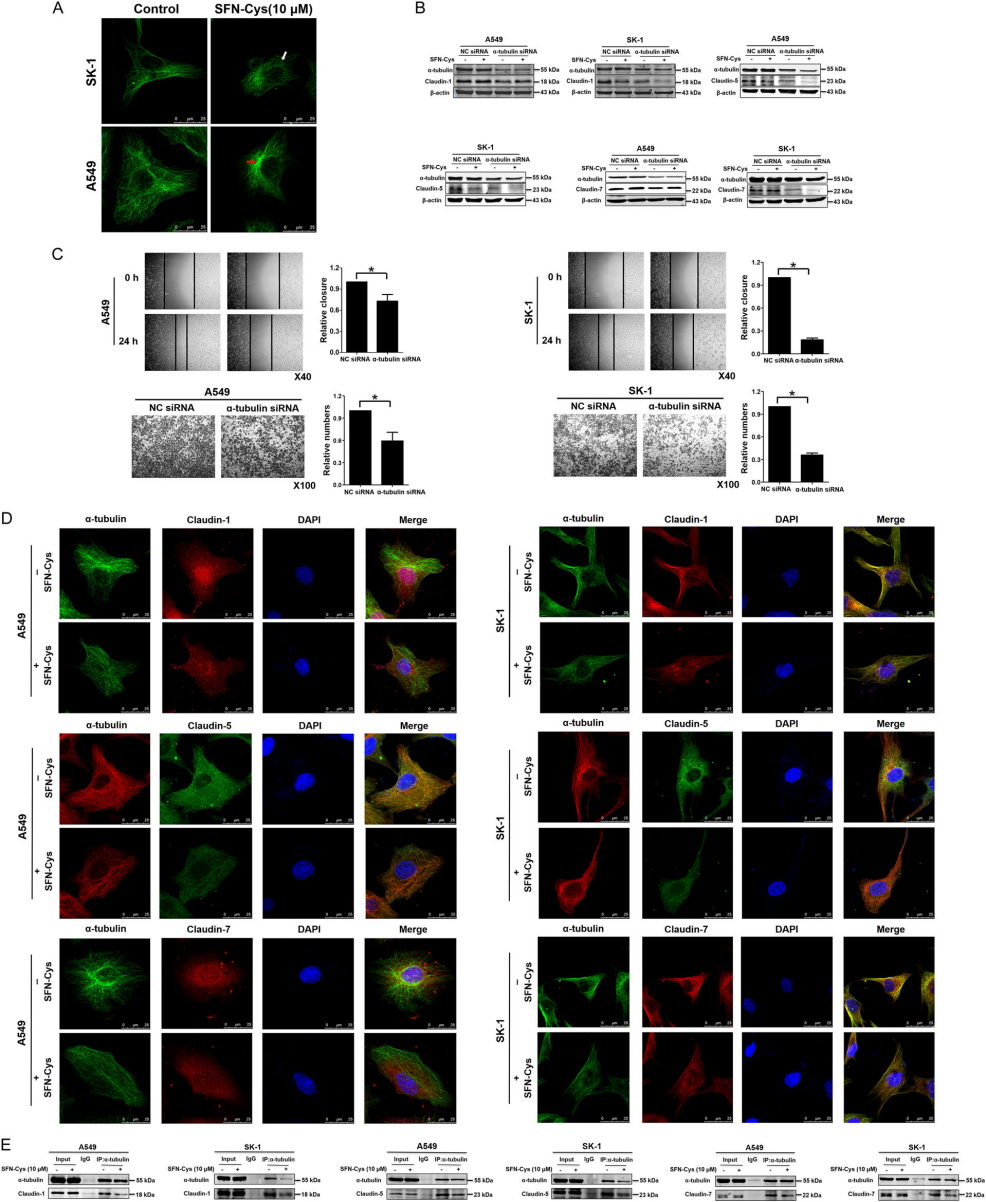
Knockdown of α-tubulin regulated the expression of Claudin-1, 5, and 7, and SFN-Cys lowered the binding of Claudin-1, 5, and 7 to α-tubulin. **a** The structure of α-tubulin was observed by immunofluorescence confocal assay with or without SFN-Cys treatment; the white arrow indicated microtubule disruption and the red arrow indicated microtubule aggregation. **b** Western blotting analysis of Claudin-1, 5, and 7 expression in the cells transfected with NC siRNA or α-tubulin siRNA. **c** Both A549 and SK-1 cells transfected with NC siRNA or α-tubulin siRNA were seeded in 24-well invasion chambers and 6-well plate to detect migration and invasiveness. **d** Immunofluorescence and confocal microscopy were employed to observe the colocalization of α-tubulin and Claudin-1, 5, and 7 in the cells. Scale bar: 25 µm. **e** After induction with 10 µM SFN-Cys for 24 h, co-immunoprecipitation was employed to detect the interaction between α-tubulin and Claudin-1 or 5, or 7, in the cells. *P < 0.05. Data were shown at means ± SD (*n* = 3)

Immunofluorescence assay showed that the distribution of α-tubulin was disrupted by SFN-Cys (Fig. 4a). In order to determine how Claudin-1 or 5, or 7, links to SFN-Cys-induced microtubule disruption, α-tubulin siRNA was used to knock down its expression. After treatment with NC siRNA and α-tubulin siRNA for 24 h, Western blotting showed that knockdown of α-tubulin decreased Claudin-1 or 5, or 7, in both A549 and SK-1 cells (Fig. 4b). Knockdown of α-tubulin suppressed significantly cell migration and invasion (Fig. 4c). Immunofluorescence confocal assay was used to examine the colocalization between Claudin-1 or 5, or 7, and α-tubulin with or without SFN-Cys. Claudins were widely distributed in whole cells just as that α-tubulin does before treatment. Meanwhile, SFN-Cys induced cell morphological changes, cells became round, cell processes became shorter, and the microtubules got disrupted and aggregated. Besides, the colocalization of Claudin-1 or 5, or 7, and α-tubulin was decreased (Fig. 4d). Co-immunoprecipitation assay revealed that SFN-Cys reduced the interaction between Claudin-1 or 5, or 7, and α-tubulin (Fig. 4e).

### SFN-NAC inhibited invasion via microtubule-mediated LC3 II accumulation

**Fig. 5:**
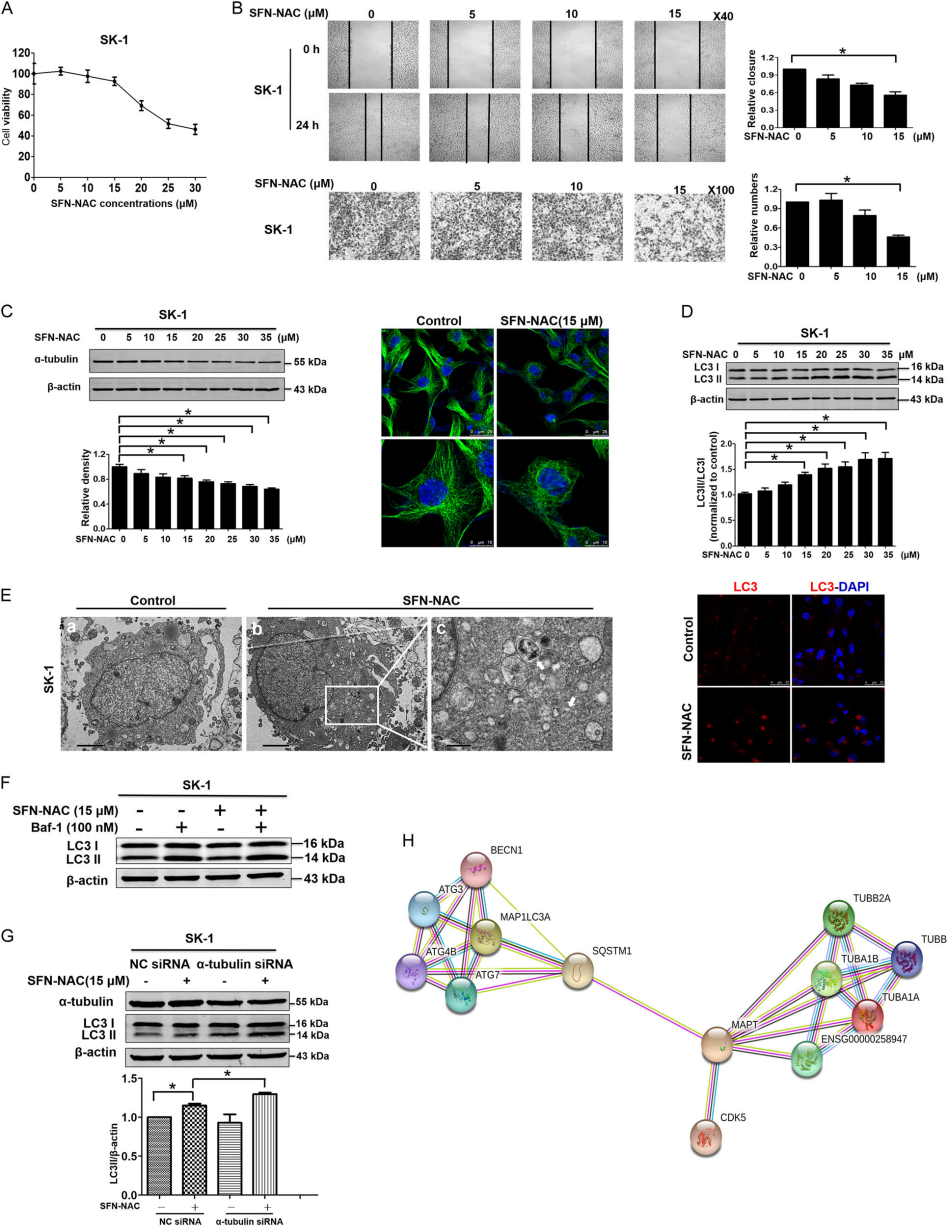
SFN-NAC inhibited invasion via microtubule-mediated LC3 II accumulation. **a** Cells were treated with various concentrations of SFN-NAC for 24 h, then cell viability was determined by Cell Proliferation Assay Kit and presented as the percentages versus the control. **b** Cells were scratched and treated with SFN-NAC at the indicated concentrations for 24 h. An image of the wound closure area was captured and measured with ImageJ software. The cells (1 × 10^5^) were seeded in 24-well invasion chambers and treated with different doses of SFN-NAC. **c** Western blotting was used to test α-tubulin expression in SK-1 cells treated for 24 h with 0, 5, 10, 15, 20, 25, 30, and 35 µM SFN-NAC. The distribution of α-tubulin was observed by immunofluorescence confocal assay with or without SFN-NAC treatment. **d** SK-1 cells were treated for 24 h with different concentrations of SFN-NAC and the expression of LC3 II and LC3 I was detected by Western blotting. **e** SK-1 cells were treated with 15 µM SFN-NAC for 24 h. Observation via transmission electron microscopy showed the autophagosomes and white arrows marked autophagosomes. Scale bar: **a**, **b**: 2 µm, **c**: 0.5 µm; fixed and stained with LC3 antibody (red) and DAPI (blue). Scale bars: 25 µm. **f** SK-1 cells co-treated with Baf-1 (100 nM) and SFN-NAC (15 µM) for 24 h, the expression of LC3 II and LC3 I was determined by Western blotting. **g** After knockdown of α-tubulin, LC3 II/LC3 I was accumulated in response to SFN-NAC. **h** The interactions among α-tubulin, autophagy-related proteins, and Tau were analyzed via the String net (TUBA1A: α-tubulin; MAP1LC3A: LC3; MAPT: Tau; SQSTM1; P62; BECN1: Beclin1; ENSG00000258947: Tubulin beta-3 chain). *P < 0.05. Data were shown as means ± SD (*n* = 3)

SK-1 cells were treated with SFN-NAC (0, 5, 10, 15, 20, 25, 30, and 35 µM) for 24 h and results showed that 15 µM SFN-NAC was the optimal concentration for migration and invasion assay (Fig. 5a). Further, after the treatment with different concentrations of SFN-NAC (0, 5, 10, and 15 µM), results showed that cell migration and invasion were significantly decreased in a dose-dependent manner (Fig. 5b). Western blotting showed that SFN-NAC downregulated the expression of α-tubulin in a dose-dependent manner. Immunofluorescence confocal assay showed that the distribution of microtubule was disrupted after treatment with SFN-NAC (Fig. 5c). Western blotting showed that SFN-NAC upregulated LC3 II/LC3 I in a dose-dependent manner (Fig. 5d). Transmission electron microscopy observation displayed autophagosomes after cells were treated by SFN-NAC for 24 h. Immunofluorescence staining results showed that the number of LC3 puncta was increased in SFN-NAC-treated cells (Fig. 5e). These results demonstrated that SFN-NAC induced the formation of autophagosome. Combined with SFN-NAC, Bafilomycin A1 (100 nM) was used to test LC3 II/LC3 I; Western blotting showed that the ratio did not change (Fig. 5f), indicating that SFN-Cys inhibited the fusion of autophagosome to lysosome. Western blotting showed that knockdown of α-tubulin induced LC3 II accumulation with the treatment of SFN-NAC (Fig. 5g), indicating that SFN-NAC inhibited the formation of autolysosome via microtubule disruption. In addition, STRING database analysis also showed that α-tubulin might interact with autophagy-related proteins via Tau (Fig. 5h).

### Knockdown of Tau reduced migration and invasion via inhibition of autolysosome formation

**Fig. 6:**
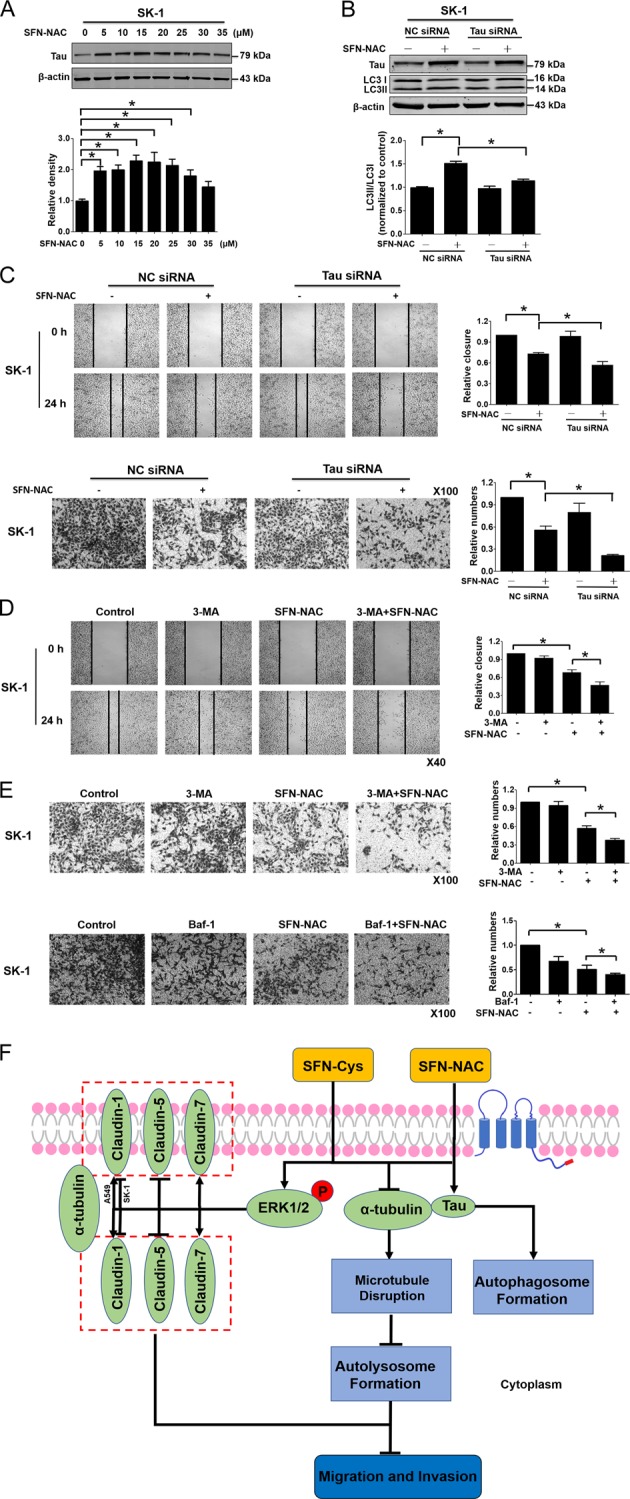
Knockdown of Tau reduced migration and invasion via inhibition of autolysosome formation. **a** SK-1 cells were treated for 24 h with increasing concentrations of SFN-NAC (0, 5, 10, 15, 20, 25, 30, and 35 µM) and the expression of Tau was determined by Western blotting. **b** SK-1 cells were transfected with Tau siRNA or control siRNA, and the expression of Tau and LC3 II/LC3 I was determined via Western blotting. **c** After transfection for 48 h, SK-1/NC siRNA and SK-1/Tau siRNA cells were plated on six-well plate and Transwell chamber at a density of 1 × 10^4^ cells per chamber, and treated with SFN-NAC (0 and 15 µM) for 24 h. Images were taken at 0 and 24 h; cells were stained with crystal violet and images were taken and analyzed. **d** SK-1 cells were scratched and treated with 2.5 mM 3-MA. SFN-NAC (0 and 15 µM) were added into the cell medium after the 2 h treatment. Images were taken at 0 and 24 h. **e** SK-1 cells were plated on Transwell chamber at a density of 1 × 10^4^ cells per chamber and treated with 2.5 mM 3-MA or Baf-1 (100 nM). SFN-NAC was added to the cell medium and treated with cells 24 h. Cells were stained with crystal violet and images were taken and analyzed. **f** A possible schematic of the involved signal pathways that sulforaphane metabolites inhibited invasion via microtubule-mediated Claudins dysfunction and inhibition of autolysosome formation in NSCLC cells. *P < 0.05. Data were shown as means ± SD (*n* = 3)

Tau was undetectable in A549 cells. SFN-NAC significantly upregulated Tau in SK-1 cells (Fig. 6a). To know the correlation of Tau to autophagy, migration, and invasion, we knocked down Tau by Tau-specific siRNA. Western blotting showed that knockdown of Tau reduced LC3 II / LC3 I after the treatment of SFN-NAC (Fig. 6b), indicating that Tau deletion inhibited the formation of autophagosome. Scratch and Transwell assay showed that SFN-NAC-induced suppression of cell migration and invasion was increased in Tau-knocked down cells (Fig. 6c). To detect that SFN-NAC-induced inhibition of autolysosome formation resulted in cell migration and invasion, we pretreated cells with autophagy inhibitor 3-MA (2.5 mM) and Bafilomycin A1 (100 nM), respectively, and then exposed to SFN-NAC; results showed that SFN-NAC-induced suppression of migration and invasion was elevated (Fig. 6d, e). These data indicated that SFN-NAC-induced inhibition of autolysosome formation reduced the migration and invasion of NSCLC cells.

In summary, we demonstrated that SFN metabolites inhibited migration and invasion via microtubule-mediated Claudins dysfunction or inhibition of autolysosome formation in NSCLC cells. Tau-mediated the formation of autophagosome might be a feedback after the cells were treated with SFN-NAC (Fig. 6f).

## Discussion

The previous studies showed that sustained phosphorylation of ERK1/2 via SFN-Cys or SFN-NAC upregulated the expression of a dozen of apoptotic proteins^12,36,44,45^. Here we found that SFN-Cys triggered ERK1/2 phosphorylation leading to microtubule-mediated Claudins dysfunction and the inhibition of migration and invasion, and SFN-NAC inhibited migration and invasion by microtubule-mediated inhibition of autolysosome formation. Although SFN-Cys and SFN-NAC triggered the different signal pathways, which did not mean that these two SFN metabolites worked differently, because we did not test both pathways for each SFN metabolite; several studies in our laboratory showed that two SFN metabolites initiated the same pathways to inhibit cancer growth and induce apoptosis^46^.

In general, Claudins are essential components of cell membrane regulating cell adhesion and motility^11^. Herein we further discovered that Claudin-1, 5, and 7 can be expressed differentially in response to SFN-Cys in the whole cells. Claudin-5 was highly expressed in lung cancer tissues and was a potential oncoprotein, which might be an ideal target for anti-cancer drug. In contrast, Claudin-7 was an anti-oncoprotein and expressed much lower in lung cancer tissues. Interestingly, SFN-Cys might decrease Claudin-5 and increase Claudin-7 levels in both A549 and SK-1 cells. Claudin-1 expression was increased in A549 cells, but decreased in SK-1 cells in response to SFN-Cys. These indicated that Claudin isotypes might have distinct roles in the different cell types. Claudin molecules might work in the opposite ways and might also collaborate to complete a certain function. Claudin-1 and Claudin-7 might form a complex with integrin α2 at the basolateral membrane of normal mouse intestine^47^. In addition, Claudin-7 was shown to be associated with CD44 in colorectal cancer cells^48^. CD44 is a surface glycoprotein to promote tumor cell motility^49^. Similar to our findings, studies showed that Claudin-11 interacted with α-tubulin to regulate cell functions^27^. Here we found that knockdown of α-tubulin downregulated Claudins, indicating that microtubule might be responsible for Claudins production and transportation, or protect Claudins via autophagosome accumulation from degrading. Here we demonstrated that Claudins worked with α-tubulin to regulate cell invasion in response to SFN-Cys, showing the complexity of cancer cell progression.

Microtubule dynamics plays a crucial role in cell morphology and movement, which is the prerequisites for cell migration and invasion. Clinical studies showed that the expression of βIII-tubulin was associated with lymphatic metastasis in NSCLC patients^50^. Importantly, depletion of βΙΙΙ-tubulin via siRNA inhibited the proliferation of tumor in mice^51^. However, our results indicated that treatment with SFN-Cys or SFN-NAC caused degradation of α-tubulin, which might induce microtubule disassembly and fragmentation, and cell morphological alteration such as cell process shortening. Microtubule-targeted drug such as paclitaxel and vinblastine killed cancer cells by binding to β-tubulin resulting in a disorder of microtubule dynamics^52^. Similar to the previous results in our laboratory, here lower concentrations of SFN metabolites also induced disequilibrium of microtubule dynamics by covalently binding to α-tubulin and decreasing the expression of α-tubulin, ultimately leading to microtubule disruption. We previously demonstrated that microtubule disruption might result from SFN metabolite-induced degradation of α-tubulin^12,36,45^. Activated proteasome might break down some damaged and misfolded cytoskeletal proteins. SFN metabolites have potentials to induce activation of the ubiquitin-proteasome system by upregulating the expression of 26S proteasome^44^. Activated Caspase3 might degrade α-tubulin and poly ADP-ribose polymerase (PARP), etc. inhibiting cell growth leading to apoptosis^15^. Tubulins are likely to interact with a number of proteins such as MAPs (microtubule-associated proteins) and Hsp70 regulating invasion. It has been reported that MAP7 domain-containing protein 3 interacted with γ-tubulin and it recruited to centrosome to promote cancer growth and metastasis^53^. Knockdown of microtubule-destabilized protein Stathmin-1 reduced lung metastasis in an orthotopic neuroblastoma mouse model^54^.

Of all MAPs, Tau showed the highest expression in NSCLC cells. Tau is also able to maintain chromosome stability by interacting with both microtubule and chromatin^55^. Here we found that knockdown of Tau augmented the SFN-NAC-caused inhibition of cell migration and invasion; meanwhile, knockdown of Tau decreased LC3 II/LC3 I in response to SFN-NAC. Therefore, as a component of cell skeleton, Tau might contribute to cell motility and promote the induction of autophagosome formation to protect cancer cells from drug attack. In the present studies, inhibition of autolysosome formation enhanced SFN-NAC-induced suppression of migration and invasion, implicating that Tau deficiency might decrease cell motility via accumulating autophagosomes and inhibiting the formation of autolysosome. Simultaneously, studies suggested that Tau deletion impaired autophagic flux in mouse, which might be due in part to microtubule changes that reduced the efficiency of autophagosome fusion with the lysosome^56^, and knockdown of Tau inhibited autophagy through regulating microtubule-based trafficking^57^.

The above-mentioned two pathway might have crosstalk in the SFN metabolite-induced inhibition of invasion. Studies showed that nitric oxide might interact with Caveolin-1 to facilitate autophagy-lysosome-mediated Claudin-5 degradation in oxygen–glucose deprivation-treated endothelial cells^58^; inhibition of autophagy might decrease the effects of nano alumina on Claudin-5 expression^59^; autophagy also enhanced tight junction barrier function and decreased Claudin-2 in intestinal epithelium cells^60^.

Collectively, SFN metabolites might inhibit invasion via microtubule-mediated Claudins dysfunction and inhibition of autolysosome formation. Investigation of SFN metabolites signaling will help us find out molecular etiology of cancer invasion and design high-efficiency drugs to treat malignant cancers.

## Supplementary information

Clinical materials (Lung adenocarcinoma)

Clinical materials (lung squamous carcinoma)
